# The effect of adipose-derived mesenchymal stem cell transplantation on ovarian mitochondrial dysfunction in letrozole-induced polycystic ovary syndrome in rats: the role of PI3K-AKT signaling pathway

**DOI:** 10.1186/s13048-024-01422-3

**Published:** 2024-04-27

**Authors:** Arash Abdi, Mina Ranjbaran, Fardin Amidi, Fariba Akhondzadeh, Behjat Seifi

**Affiliations:** 1 Department of Physiology, School of Medicine, Tehran University of Medical Sciences, Tehran, Iran; 2 Department of Anatomy, School of Medicine, Tehran University of Medical Sciences, Tehran, Iran; 3 Department of Physiology, School of Medicine, Shahid Beheshti University of Medical Sciences, Tehran, Iran

**Keywords:** Mesenchymal stem cells, PCOS, Oxidative stress, Inflammation, PI3K-AKT, Mitochondrial dynamic, LY294002

## Abstract

**Objective:**

The present study aimed to elucidate how mesenchymal stem cells (MSCs) application could efficiently attenuate pathological changes of letrozole-induced poly cystic ovary syndrome (PCOS) by modulating mitochondrial dynamic via PI3K-AKT pathway.

**Methods:**

Thirty-two female rats were randomly divided into four experimental groups: Sham, PCOS, PCOS + MSCs, and PCOS + MSCs + LY294002. The Sham group received 0.5% w/v carboxymethyl cellulose (CMC); the PCOS group received letrozole (1 mg/kg, daily) in 0.5% CMC for 21 days. Animals in the PCOS + MSCs group received 1 × 10^6^ MSCs/rat (i.p,) on the 22th day of the study. In the PCOS + MSCs + LY294002 group, rats received LY294002 (PI3K-AKT inhibitor) 40 min before MSC transplantation. Mitochondrial dynamic gene expression, mitochondrial membrane potential (MMP), citrate synthase (CS) activity, oxidative stress, inflammation, ovarian histological parameters, serum hormone levels, homeostatic model assessment for insulin resistance (HOMA-IR), insulin and glucose concentrations, p-PI3K and p-AKT protein levels were evaluated at the end of the experiment.

**Results:**

PCOS rats showed a significant disruption of mitochondrial dynamics and histological changes, lower MMP, CS, ovary super oxide dismutase (SOD) and estrogen level. They also had a notable rise in insulin and glucose concentrations, HOMA-IR, testosterone level, tumor necrosis factor-α (TNF-α) and interleukin-6 (IL-6) levels, ovarian malondialdehyde (MDA) content as well as a notable decrease in p-PI3K and p-AKT protein levels compared to the Sham group. In the PCOS + MSCs group, the transplantation of MSCs could improve the above parameters. Administration of LY294002 (PI3K-AKT pathway inhibitor) deteriorated mitochondrial dynamic markers, oxidative stress status, inflammation markers, hormonal levels, glucose, and insulin levels and follicular development compared to the PCOS + MSCs group.

**Conclusions:**

This study demonstrated that the protective effects of MSC transplantation in regulating mitochondrial dynamics, promoting mitochondrial biogenesis, competing with redox status and inflammation response were mainly mediated through the PI3K-AKT pathway in the PCOS model.

**Supplementary Information:**

The online version contains supplementary material available at 10.1186/s13048-024-01422-3.

## Introduction

Polycystic ovary syndrome (PCOS) is a common and intricate endocrine disorder and metabolic condition, making it one of the most prevalent endocrine disorders among women of reproductive age. It affects approximately 8–13% of this demographic worldwide [1]. Current treatment strategies for PCOS patients are often unsatisfactory and this syndrome accounts for primary infertility in women [2]. Typical symptoms that concern patients with PCOS include hirsutism, irregular and chronic ovulation, acne, oily skin, oligomenorrhea, alopecia, insulin resistance, dyslipidemia and hyperandrogenism [3]. Irregular and chronic ovulation is a critical reason for infertility in patients with PCOS. Even though the pathogenesis and etiology of PCOS are not yet fully understood [4], studies suggest that its pathogenesis is influenced by various mechanisms including, genetic factors, oxidative stress, inflammation, lipid imbalance, and insulin resistance (IR) [5].

PCOS, one of the considerable diseases that restrict reproductive outcome, has been vigorously characterized by increased inflammation, oxidative stress, mitochondrial dysfunction, detrimental modification in cell signaling pathways (such as phosphoinositide-3-kinase-protein kinase B/AKT or PI3K-AKT), hyperinsulinemia and infertility [6]. The women with PCOS often have complication in metabolism of androgen and estrogen. This results in enhanced concentration of androgens (hyperandrogenism) and that means high levels of androgens account for ovulation disruption and effects of quality and fertility of oocyte in women with PCOS [7]. The PI3K/AKT signaling pathway is a critical component of insulin signaling, contributing significantly to not only insulin resistance but also playing a crucial role in various processes such as cell proliferation, growth, migration, invasion, inhibition of apoptosis, and angiogenesis [8, 9]. Modulating PI3K-AKT signaling pathway may cause significant changes in granulosa cell proliferation, follicular development and oocyte fertility [10]. Recent research indicates that inhibiting the PI3K-AKT pathway may impact insulin functionality and glucose metabolism [11]. Consequently, the PI3K-AKT signaling pathway has essential role in the pathogenesis PCOS disorders [12].

Mitochondria is a dynamic organelle associated with a delicate balance between fission and fusion [13]. Disturbance in mitochondrial dynamics, genes expression, enzymes and mitochondrial membrane potential (MMP) can lead to inordinate between fusion and fission, and occurrence various disorders such as neurodegenerative, cardiovascular, renal and reproductive diseases [14]. There is growing evidence showing that dysregulation of mitochondrial dynamic function is an essential cause of PCOS development and decreased fertility in women [15]. It has been reported that tissues exhibiting IR are affected by a decline mitochondrial content, gene expression and enzyme activity. In this context, hyperandrogenism, insulin resistance, oxidative stress, and glucose intolerance are exerted by alteration in mitochondrial function and biogenesis [16]. Oxidative stress, established by an imbalance between antioxidant and pro-oxidant factors, can cause abnormal granulosa cells function and negative fertility outcome [16].

Nowadays, the therapeutic options for PCOS patients are limited. The guidelines of PCOS by the World Health Organization (WHO) recommend lifestyle changes, including dietary modification, exercise and use of drugs such as metformin to alleviate hyperandrogenism and metabolic symptoms [17]. Using novel techniques, such as gene or cell therapy, may present an achievable way to treat PCOS.

Over the last decade, stem cell-based treatments have garnered considerable interest as a potential new therapeutical strategy for different endocrine disorders, such as PCOS [18–20]. Among the stem cells used, mesenchymal stem cells have garnered growing attention due to their potential therapeutic applications in various disorders of the female reproductive system. MSCs have received notable interest in research investigations due to their specific biological function, ease of isolation, anti-inflammatory, anti-apoptotic, antioxidant, and immunomodulatory effects, making them good candidate for cell therapy [21, 22]. The positive effects of MSCs are attributed to their ability to differentiate into epithelial, endothelial and stromal cells, regulate immune responses, and modulate paracrine signaling pathways [23]. Interestingly, recent findings have revealed that MSCs restore injured tissues and cells by delivering their mitochondria [24]. Xie et al., demonstrate that MSCs by activating PI3K-AKT pathway significantly can improve adrenal cells proliferation and differentiation [25]. The survival of follicles in the ovary related to balanced PI3K-AKT pathway [26]. MSCs can improve ovarian function by activating the PI3K-AKT pathway via a paracrine mechanism [27]. In a study conducted by Ataf et al., only fission and fusion parameters were evaluated, and mitochondrial membrane potential and biogenesis were not assessed [28]. Safai's et al., research evaluated the effect of vitamin D only on mitochondrial biogenesis within a model of polycystic ovary syndrome induced by DHEA, without assessing the complexities of mitochondrial dynamics [29]. In another study, only mitochondrial dynamics were investigated in the polycystic ovary syndrome rat model [30]. Xie et al. demonstrated that transplantation of mesenchymal stem cells could enhance ovarian function by decreasing systemic and local inflammation. However, their study did not assess the effect of mesenchymal stem cells on the mitochondrial function of ovarian tissue [31]. The novelty aspect of our study is evaluating effects of MSCs transplantation on mitochondrial dynamics, biogenesis, and function in ovaries via the PI3K-AKT pathway in letrozole-induced polycystic ovary syndrome. In this current investigation, we assessed the effects of MSCs transplantation on mitochondrial dynamic, biogenesis, oxidative stress, inflammation, IR, hormonal assay and histopathological change of ovarian tissue via PI3K-AKT pathway in letrozole induced PCOS rats. We hypothesized that PCOS induction impairs mitochondria dynamics in the ovary, and transplanted MSCs are capable of attenuating the complication of this syndrome and rescue ovarian cells by restore mitochondria dynamic and biogenesis, mainly through the PI3K-AKT pathway.

## Materials & methods

### In-vitro study

#### Culture and isolation of rat adipose tissue-derived MSCs

Six Wistar rats were given ketamine and xylazine anesthesia before their epididymal adipose tissue was obtained for MSCs collection under sterile conditions. Epididymal fat tissue was chosen based on ethical consideration in animal research, the availability of this tissue and the survival of the rats. The adipose tissues were finely cut into tiny pieces and then incubated for 15 min at 37 ˚C with a moderate agitation in collagenase Type I liquid (Invitrogen Gibco) at a final concentration of 0.1% [32, 33]. The mesenchymal stem cell pellet from adipocytes was separated from digested mixture by centrifuging it for 15 min at 1500 rpm, then diluted with 4 ml of Dulbecco’s modified Eagle’s medium (DMED) containing 15% fetal bovine serum (FBS). After disposing the supernatant, the cellular pellet was filtered through a nylon mesh filter with a pore size of 200 µm for remove any remaining undigested tissues. A portion of the cell suspension was then cultivated in DMEM-High Glucose with streptomycin (100 µg/ml), 15% FBS and penicillin (100 U/ml). The mixture was then incubated at 37 ˚C with 5% CO2 and 95% humidity. Approximately 2 days after the start of cell culture, the first medium was changed, and non-attached cells were removed. The medium was changed every 48 or 72 h. When MSCs reached 80–90% confluency, they were incubated with ethylenediaminetetraacetic acid (EDTA) for fresh passage and cultured for 2 passages (Figure S1) [32–34].

#### Characterization of MSCs by flow cytometric analysis

We isolated these MSCs and at the second passage, MSCs were evaluated using a BD FACSTM Calibur flow cytometer for flow cytometric analysis (Figure S2).

#### Characterization of MSCs by Differentiation Assay

The capability of MSCs to differentiate into osteocyte and adipocyte lineages was assessed during the second passage.

#### Osteogenic differentiation

MSCs were plated at a density of 1 × 10^4^ cells per well in 24-well plates (SPL, Korea) and incubated at 37 °C for 24 h. Osteogenic differentiation media, consisting of 100 mM dexamethasone, 10 mM β-glycerophosphate, and 5 μg/mL ascorbic acid, was added every 72 h over a period of 3 weeks. Subsequently, the cells were fixed using 4% paraformaldehyde, and mineralization was assessed through Alizarin Red S staining (Figure S3a) [34, 35].

#### Adipogenic differentiation

MSCs at a density of 15 × 10^3^ cells per well, were cultured in 24-well plates (SPL, Korea) and maintained at 37 °C. Following a 24-h incubation, adipogenic differentiation media (containing 100 mM indomethacin, 0.5 mM 3-isobutyl-methylxanthine, 250 mM dexamethasone, and 5 mM bovine insulin) was introduced to the cells every 3 days for a duration of 2 weeks. Subsequently, the cells were fixed with 4% paraformaldehyde, and the presence of adipose vacuoles was identified through Oil Red O staining (Figure S3b) [34, 35].

### In-vivo study

#### Animals

For this study, we acquired thirty-two female Wistar rats with weights ranging from 180 to 200 g from the Department of Animal Research at the School of Medicine, Tehran University of Medical Sciences. These rats were housed in a controlled laboratory environment, with a 12-h dark–light cycle, temperature maintained at 22 ± 2 ºC, humidity kept between 30–40%, and they had free access to water and food. All experimental procedures were duly approved by the Ethics Committee of Tehran University of Medical Sciences (Project number: 54115, ethics committee reference number: IR.TUMS.NI.REC 1400.957).

**Fig. 1:**
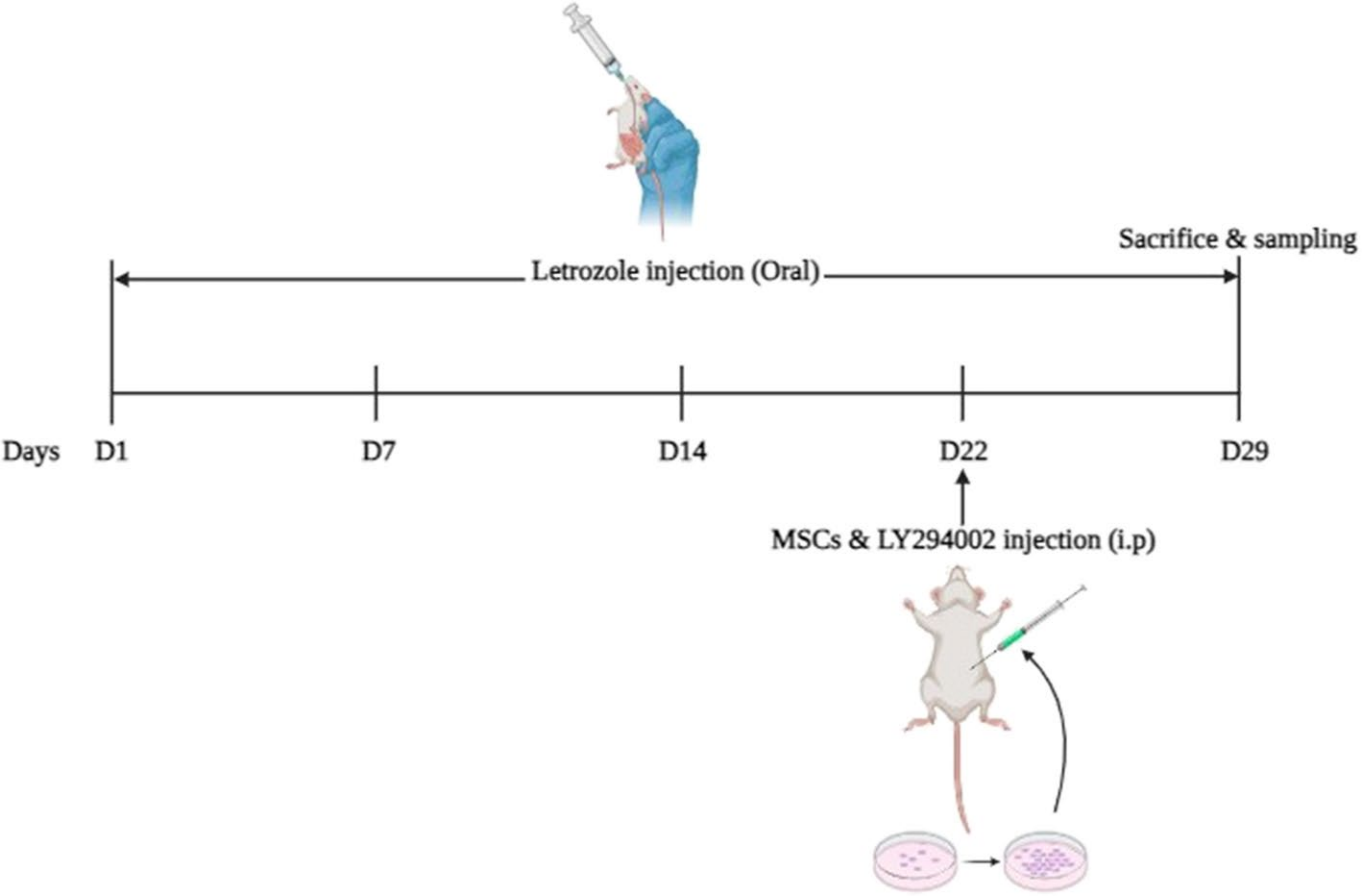
Schematic figure of the experimental study, illustrating the time and duration of drug administration

In all animals, vaginal smears were performed for 6 constitutive days before the induction of PCOS and stained with crystal violet (Tebvaran, Iran) so as to determine the estrous cycle by light microscopy. Rats that exhibited proestrous, estrus, meta-estrus and di-estrus cycles were included in the study. The animals were distributed randomly into 4 groups eight: (1) Sham (2) PCOS (3) PCOS + MSCs (4) PCOS + MSCs + LY294002. Rats in the Sham group were given oral 0.5% CMC solution for 21 consecutive days and were scarified on 29^th^ day of the study. In the PCOS group, polycystic ovary model was induced by intragastric administration of 1mg/kg letrozole (Aboreihan daru, Iran) dissolved in 0.5% w/v carboxymethylcellulose (CMC) for 21 consecutive days and rats were observed until the 29th day of the study [28]. In the PCOS + MSCs group, the procedure was identical to the PCOS group, except that MSCs (1 × 10^6^ cells, at passage 2, suspended in 50 µl saline) were transplanted intraperitoneally on the 22nd day of the study. The PCOS + MSCs group also received an intraperitoneal injection of dimethyl sulfoxide (DMSO) (2ml/kg) 40 min before MSC transplantation to control for off-target toxic effects [20]. For the PCOS + MSCs + LY294002 group, the method was identical to the PCOS rat plus intraperitoneal injection of LY294002 dissolved in DMSO (15mg/mL ly294002 in DMSO at concentration of 30 mg/kg) on the 22nd day of the study 40 min before MSCs transplantation [36]. To prevent the autonomous restoration of ovaries, we continued oral administration of letrozole following the transplantation of MSCs treatment for a duration of 7 days in the PCOS + MSCs and the PCOS + MSCs + LY294002 groups (Fig. 1).

Furthermore, this study includes two additional groups: Sham + LY294002 and PCOS + LY294002. Rats in the Sham + LY294002 group underwent the Sham procedure and were administered an intraperitoneal injection of LY294002 dissolved in DMSO (15mg/mL ly294002 in DMSO at concentration of 30 mg/kg) on the 22nd day. For the PCOS + LY294002 group, the method was the same to the PCOS + MSCs + LY294002 group, without MSCs administration.

On the 29^th^ day of the study, animals were anesthetized through an intraperitoneal injection of ketamine (100 mg/kg) and xylazine (10 mg/kg). They were then positioned in a supine posture, and a vertical midline incision was performed on their abdominal area. The ovaries of the animals were carefully removed from the body and washed in normal saline to drain the blood, and the excess fat was carefully removed without damage to the ovarian tissue, then the weight and volume of the ovaries were determined. The left ovary was rapidly frozen in liquid nitrogen to evaluate oxidative stress parameters, assess mitochondrial dynamics, and determine the levels of p-AKT and p-PI3K proteins. The right ovary was preserved in a 10% formalin solution for subsequent histological examination. For biochemical analysis, blood samples were obtained from the inferior vena cava, centrifuged at 4000 g for 10 min at 4°C, and the serum was collected and stored at -70°C until further analysis.

#### Determination of the estrous cycle

**Fig. 2:**
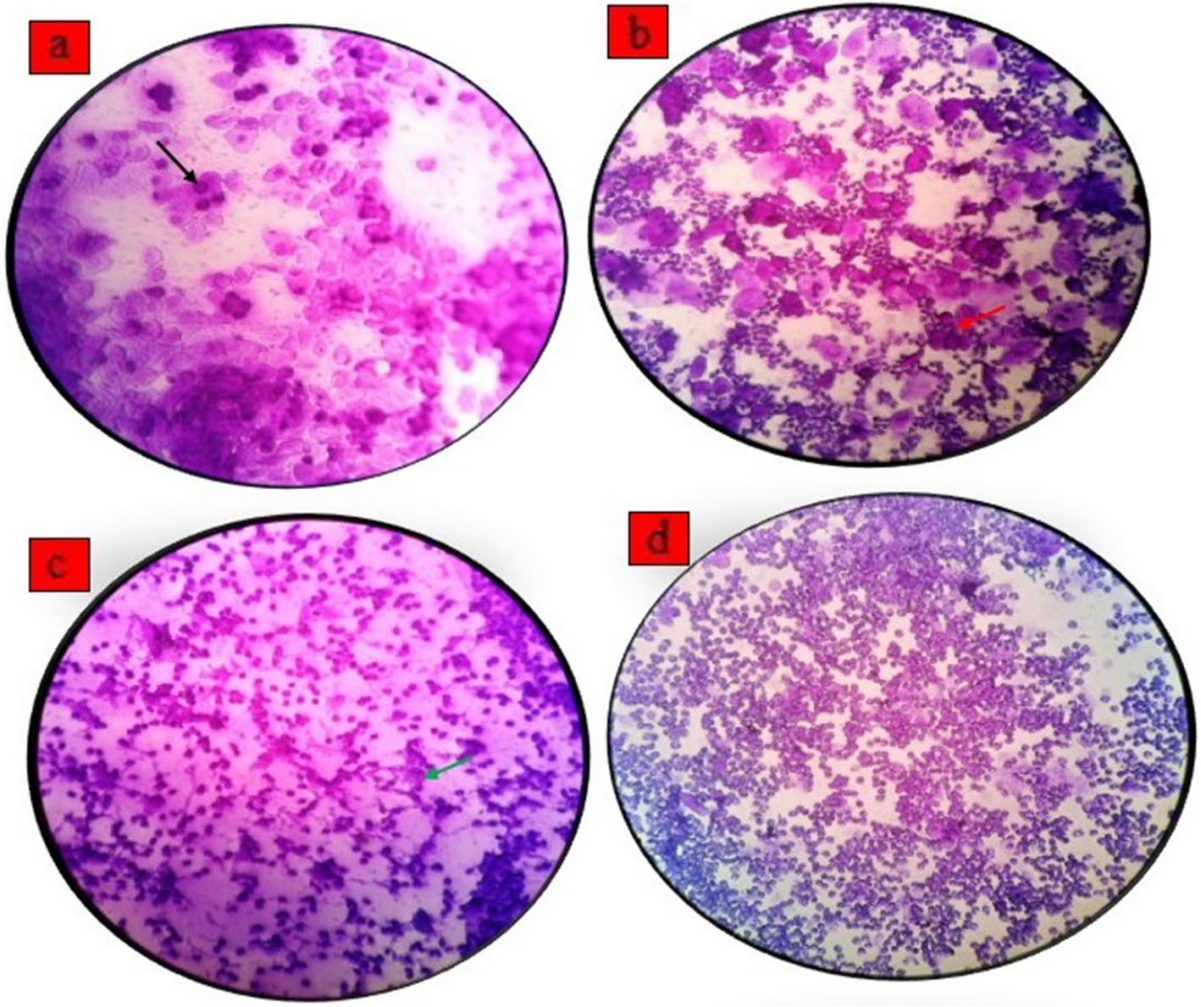
The cytological assessment of vaginal smears during different stages of the estrous cycle. To identify rats in proestrus (**a**), estrus (**b**), metestrus (**c**), or diestrus (**d**), we identified three cell types in the vaginal smear samples: nucleated epithelial cells (indicated by black arrows), cornified squamous epithelial cells (indicated by red arrows), and leukocytes (indicated by green arrows). The magnification used was × 40

The rats' estrous cycle was assessed by collecting vaginal epithelial cell smears using the following procedure: initially, 100 μL of sterile saline was gently poured into the vaginal cavity and, after a 10-s interval, aspirated from the pipette tip. This process was repeated 5 to 6 times. Subsequently, the fluid containing a few drops of cell suspension was deposited onto a glass slide, air-dried, and then stained with 0.1% crystal violet. Finally, the stained slides were examined under a light microscope at a magnification of 40 × (Fig. 2).

#### Evaluation of the body and ovarian weight, length and width

Final body mass and ovarian weight were determined using a digital scale, the ovary length and width were assessed by a vernier caliper.

#### Measurement of MDA content and SOD activity in ovarian tissue

The level of MDA was determined following the esterbauer and cheesman methods. MDA reacts with thiobarbituric acid (TBA) to form a pink pigment. To do this, 50 mg of ovarian tissue was homogenized with 1 ml of a 10% trichloroacetic acid (TCATM; Sigma, USA) solution to precipitate the proteins. After centrifugation at 3000 rpm (Falcone, USA) for 10 min, 500 μl of the resulting solution was extracted, and 500 μl of a 0.67% thiobarbituric acid (TBATM; Sigma, USA) solution was added to it [37]. The mixture was then heated in a boiling bath for 10 min. Following cooling, the absorbance of this solution was read at 532 nm, and its concentration was calculated by comparing it with a standard curve [38].

To evaluate the activity of the SOD enzyme, we used an ELISA kit from Navid Salamat CO, Iran. A total of 100 mg of ovarian tissue was mixed with 500 μl of lysis buffer and homogenized. The solution was then centrifuged at 4 °C, and the resulting supernatant was used to determine the SOD activity using the kit.

$$\mathrm{SOD\; activity }\left({\text{U}}/\mathrm{ml\; or\; mg\; protein}\right)=\mathrm{OD\; Test}/\mathrm{OD\; Control}\times 200$$

#### Measurement of TNF-α and IL-6 levels in ovarian tissue

Ovarian tissue samples (50 mg) were homogenate in 1 ml of 1X PBS and kept frozen at -20 degrees Celsius overnight. The cell membranes were disrupted by performing two freeze–thaw cycles, and the homogenates were then centrifuged at 5000 g for 5 min at 2–8 degrees Celsius. The supernatant was separated and analyzed according to the manufacturer's instructions (CUSABIO, USA) to measure the levels of TNF-α and IL-6 in the ovarian tissue tissues. The optical density of each well was assessed using a microplate reader (BioTek Instrument, ELX 800, USA) with a wavelength of 450/570 nm.

#### Western blotting

Western blot evaluation was performed as described previously with some modifications [39]. For this assessment, ovarian tissue was lysed with RIPA buffer. After 20 min of incubation at 4°C, the lysates were centrifuged for 20 min at 14,000 rpm. The protein concentration was assessed by using the Bradford Protein Quantification kit (DB0017, DNAbioTech, Iran) following the manufacturer's guidelines. The tissue lysates were combined with an equal amount of 2X Laemmli sample buffer. Following a 5 min boiling period, lysates (20 μg) were then processed through SDS-PAGE and subsequently moved onto a 0.2 μm immune-Blot™ polyvinylidene difluoride (PVDF) membrane (Cat No: 162–017777; Bio-Rad Laboratories, CA, USA). The membranes were later blocked using 5% BSA (Cat No: A-7888; Sigma Aldrich, MO, USA) in 0.1% Tween 20 for 1 h. Afterward, they were exposed to Anti-p-AKT1 (Cat No: ab278565, abcam), Anti-p-PIK3 (Cat No: ab182651, abcam), and anti-β actin-loading control antibodies (Cat No: ab8227, Abcam) for 1 h at room temperature. Following this, the membranes underwent a series of three washes with TBST, and were then subjected to incubation with goat anti-rabbit IgG H&L (HRP) (Cat No: ab6721; Abcam) secondary antibody. Enhanced chemiluminescence (ECL) was used for 1–2 min on the membranes, and subsequently, protein expression was standardized to β-actin. The densitometry analysis of protein bands was carried out utilizing gel analyzer Version 2010a software (NIH, USA). Finally, the percentage area under the curve for each band was divided by the percentage area under the curve of its corresponding actin band, and these calculated values were compared between groups as previously detailed [40].

#### Histopathological procedure

Ovarian samples were first preserved in a 10% formalin solution, then embedding them in paraffin blocks and slicing them into 5 µm-thick sections. These sections were then subjected to hematoxylin and eosin (H & E) staining to examine alterations in preantral, antral, cystic follicles, and corpus luteum using a light microscope [41].

### Mitochondrial function assay

#### Mitochondrial membrane potential (MMP)

The ovarian tissue was incubated to JC-1 dye (Catalog number: T4069) at a concentration of 2 µM for a duration of 30 to 60 min at 37 °C in the absence of light. Afterward, it was rinsed three times with Phosphate-buffered saline (PBS). A mixture of glycerol and PBS was applied to the sample, and once a coverslip was positioned, the sample was photographed using a fluorescent microscope (Olympus).

#### Measurement of mitochondrial citrate synthase (CS) activity assay

Initially, 100 mg of ovarian tissue was placed in 1 ml of Ripa buffer (Catalog number: DB9719). The samples were homogenized under refrigeration and subsequently centrifuged at 4000 rpm for 20 min. Following this, 40 µl of the supernatant and 50 µl of the standards were poured into separate wells. Then, 50 µl of Streptavidin HRP was added. Subsequently, 10 ml of antibody was applied to the sample wells. After a 1h incubation, the solution within the wells was discarded. A washing solution was then added to the wells, left for 30 s, and then emptied. This washing procedure was repeated four times. Once the washing and drying steps were completed, 50 µl of chromogen A and subsequently 50 µl of chromogen B solutions were added to the wells. The plate was gently agitated and left to incubate in the dark at a temperature of 37 ºC for 10 min. After this incubation period, stop solution was added to all the wells until the blue color transformed into yellow. The absorbance of the wells was measured at 450 nm using an ELISA reader.

#### Quantitative real-time PCR for fission and fusion genes

Ovarian tissue samples were subjected to RNA extraction using the Gene histogene RNA purification kit following the kit's guidelines (Histogene.ir, Iran). Subsequently, the RNA was reverse transcribed into cDNA using the revert Aid H Minus Reverse Transcriptase [42]. Finally, cDNA was applied as a template to detect nuclear factor erythroid 2–related factor 2 (NRF1), Mitofusin 2 (Mfn2), Dynamin-related protein 1 (DRP-1), Peroxisome proliferator-activated receptor-γ coactivator (PGC-1) and the mitochondrial transcription factor A (TFAM) gene expressions by real-time PCR system. The primer sequences were listed as follows: **TFAM gene**, F (5´- CGA GGT CTT TTT GGT TTT CC-3´); R(5´-AAG GTG TAT GAA GCG GAT TTT-3´), **NRF1 gene**, F (5´-GAAAACGACAGAAACCTCCATC-3´); R(5´-CTCCATCCTCCCGAACCT-3´), **PGC-1 gene**, F(5´-AACAAGCACTTCGGTCATCC-3´); R(5´-CTTCGCTGTCATCAAACAGG-3´), **MFN2 gene,** F(5´-AGTCAACACCATCAGTAGCCA-3´); R(5´-CTTGAGAGGGGAAGCATTCAC-3´) and **DRP-1 gene**, F(5´- CCTCAGATTGTCGTAGTGGGA-3´); R(5´-CCCCATTCTTCTGCTTCAACT-3´). The relative gene expressions were evaluated by 2^−ΔΔCt^ method.

#### Fasting blood glucose level, insulin level, Homeostatic Model Assessment for Insulin Resistance (HOMA-IR), insulin sensitivity index (ISI)

Fasting blood glucose levels were assayed by the enzymatic calorimetric glucose oxidase method. Serum insulin level was determined using ELISA kit (Padgin Teb Co, Iran).

Insulin resistance (IR) was assessed by using the Homeostatic Model Assessment for Insulin Resistance (HOMA-IR index) based on Matthews's formula [43], which can be expressed as follows:

$$\mathrm{Fasting\; glucose }\;({\text{mmol}}/{\text{l}}) \times \mathrm{ Fasting\; insulin }\;({\text{mIU}}/{\text{I}})/22.5$$

We additionally assessed the insulin sensitivity index (ISI) value in rats as follows [44]:

$${\text{ISI}}=\mathrm{ ln }\;(1/\mathrm{ Fasting\; glucose }\times \mathrm{ Fasting\; insulin})$$

#### Hormonal assay

Serum steroid and testosterone hormones were assayed using an ELISA kit (Padgin Teb Co, Iran). All procedures were carried out according to the manufacturer’s instructions.

#### Statistical analysis

Data analysis was conducted by using GraphPad Prism 9.0 statistical software. The data were presented as the mean ± SEM. To assess the variables, a one-way ANOVA was employed, followed by Turkey's post hoc test to compare various groups. A statistical significance level of *p* < 0.05 was considered for determining statistical significance.

## Results

### Effects of adipose-derived MSC and LY294002 administrations on body weight, ovarian weight, length, and width

Table 1 illustrates that the PCOS group exhibited an increase in body weight in comparison with the Sham group, while the administration of MSCs prevented weight gain in the animals. The effects of MSCs on body weight were eliminated by LY294002 administration.

**Table 1:** Body and ovarian weight and ovarian length and width in different group

Experimental groups				
Parameters	Sham	PCOS	PCOS + MSCs	PCOS + MSC + LY294002
Final body weight (g)	179 ± 2.1	218 ± 4.6^***^	196.6 ± 3.41^##**^	221 ± 5.68^$$$**^
Ovarian weight (g)	0.04 ± 0.001	0.72 ± 0.006^***^	0.15 ± 0.001^##**^	0.74 ± 0.003^$$$***^
Ovarian length (mm)	5.37 ± 0.25	7.68 ± 0.26^***^	5.77 ± 0.23^##*^	7.80 ± 0.26^$$$**^
Ovarian width (mm)	4.169 ± 0.20	7.25 ± 0.25^***^	4.99 ± 0.20^##**^	7.54 ± 0.24^$$$***^

Data are presented as mean ± SEM

^*^p < .05

^**^p < .01

^***^p < .001 significant differences versus the sham group

^##^*p* < .01 significant differences versus the PCOS group

^$$$^*P* < 0.001 significant differences versus the PCOS + MSCs group

PCOS resulted in significant increases in ovaries weight, length and width compared to the Sham group. MSCs administration significantly preserved these parameters compared to the PCOS group. LY294002 administration significantly omitted the effects of MSCs on the ovaries weight, length and width compared to the PCOS + MSCs group (Table 1).

The administration of LY294002 in the Sham + LY294002 and PCOS + LY294002 groups had no effect on body weight (Figure S4a), ovarian weight (Figure S4b), length (Figure S4c) and width (Figure S4d).

### Effects of adipose-derived MSC and LY294002 administrations on cystic follicles, corpora lutea, preantral follicles and antral follicles

**Fig. 3:**
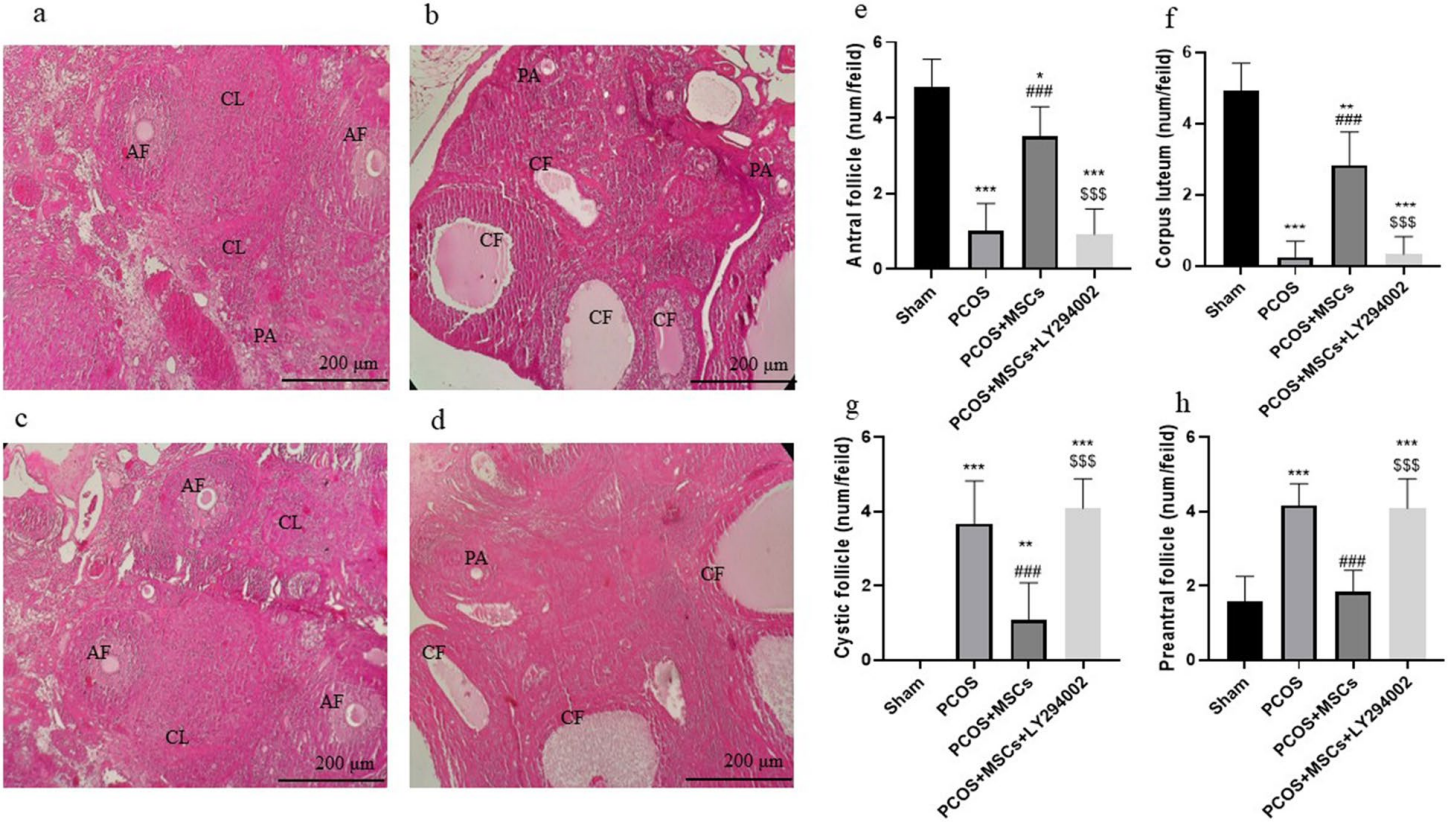
Histopathological examination of ovarian tissue. Sham group (**a**), PCOS group (**b**), PCOS + MSCS (**c**), PCOS + MSCs + LY294002 (**d**), antral follicle (**e**), corpus loteum (**f**), cystic follicle (**g**) and preantral follicle (**h**). Data are presented as means ± SEM. * *P* < 0.05, ** *P* < 0.01 and *** *P* < 0.001 significant differences versus the Sham group, ## *P* < 0.01 and ### *P* < 0.001 significant differences versus the PCOS group. $$$ *P* < 0.001 significant differences versus the PCOS + MSCs group. e: antral follicle, f: corpus loteum, g: cystic follicle, and h: preantral follicle. Severe histological changes were observed including cystic follicles (CF), corpus luteum (CL), preantral follicles (PA) and antral follicles (AF)

Figure 3 shows the histological examination of ovarian tissue (a-d) and the number of ovarian cystic follicles and different types of follicles in all groups (e–h). As shown in the Fig. 3e and f, the numbers of antral follicles and corpus lutea formed in the ovary significantly decreased in the PCOS group compared to the Sham group (*p* < 0.001). Our results indicated that treatment with MSCs markedly restored the numbers of antral follicles and corpus lutea compared to the PCOS group (*p* < 0.001). Figure 3g and h show the number of formed cystic and preantral follicles increased in the PCOS group compared to the Sham group. Our findings demonstrated that administration of MSCs significantly decreased the numbers of formed cystic and preantral follicles compared to the PCOS group (*p* < 0.001). The administration of LY294002 markedly elevated the number of formed cystic and preantral follicles in comparison with the PCOS + MSCs group (*p* < 0.001).

In the Sham + LY294002 and PCOS + LY294002 groups, the administration of LY294002 (PI3K-AKT inhibitor) had no significant effect on mentioned follicular parameters (Figure S5).

### Effects of adipose-derived MSC and LY294002 administrations on parameter of oxidative stress (MDA level and SOD activity) and inflammation markers (TNF-α and IL-6 levels) in the ovaries

**Fig. 4:**
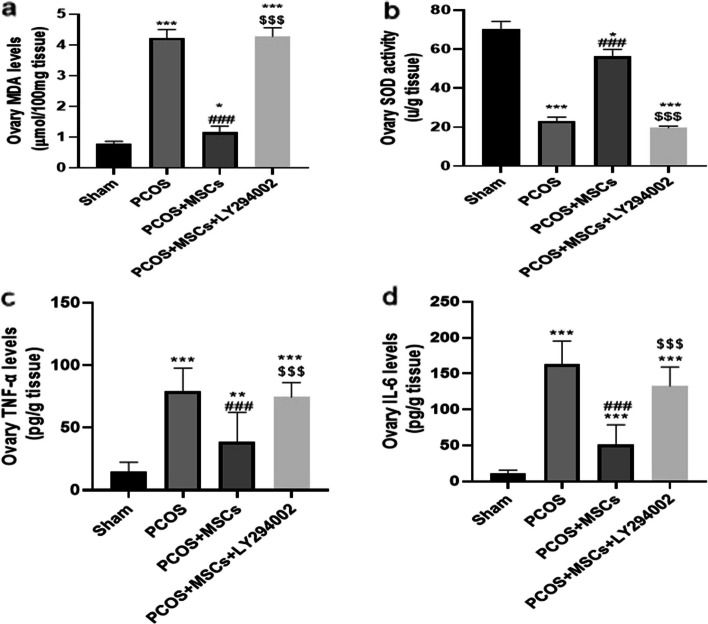
Changes in ovarian MDA level (**a**), SOD activity (**b**), TNF-α (**c**) and IL-6 (**d**) levels among the different groups. Data are presented as mean ± SEM. ^*^p < .05 and ^***^p < .001 significant differences versus the Sham group. ^###^*p* < .001 significant differences versus the PCOS group. $$$ *P* < 0.001 significant differences versus the PCOS + MSCs group. (MDA: Malondialdehyde, SOD: Superoxide dismutase, TNF-α: tumor necrosis factor, IL-6: interlukin-6)

PCOS induction caused a significant increase in ovarian MDA levels compared to the Sham group (Fig. 4a, *P *< 0.001). MSCs administration decreased ovarian MDA level compared to the PCOS group (*P* < 0.001). However, the MDA level increased remarkably in the PCOS + MSCs + LY294002 group compared to the PCOS + MSCs group (*P* < 0.001).

PCOS caused a significant decrease in ovarian SOD activity compared to the Sham group (Fig. 4b, *P*< 0.001). The administration of MSCs elevated SOD activity in ovarian tissue compared to the PCOS group (*P* < 0.001). However, SOD activity was markedly reduced in the PCOS + MSCs + LY294002 group compared to the PCOS + MSCs group (*P* < 0.001).

The administration of LY294002 in the Sham + LY294002 and PCOS + LY294002 groups did not cause a significant alteration in MDA level (Figure S6a) and SOD activity (Figure S6b).

In the PCOS group, ovarian levels of TNF-α and IL-6 significantly increased compared to the sham group (Fig. 4c and d, *P* < 0.001). MSCs administration significantly prevented the rises in ovarian TNF-α and IL-6 in comparison with the PCOS group (*P* < 0.001). However, TNF-α and IL-6 levels notably elevated in the PCOS + MSCs + LY294002 group compared to the PCOS + MSCs group (*P* < 0.001).

### Effects of adipose-derived MSC and LY294002 administrations on mitochondrial dynamic markers, MMP and CS activity

**Fig. 5:**
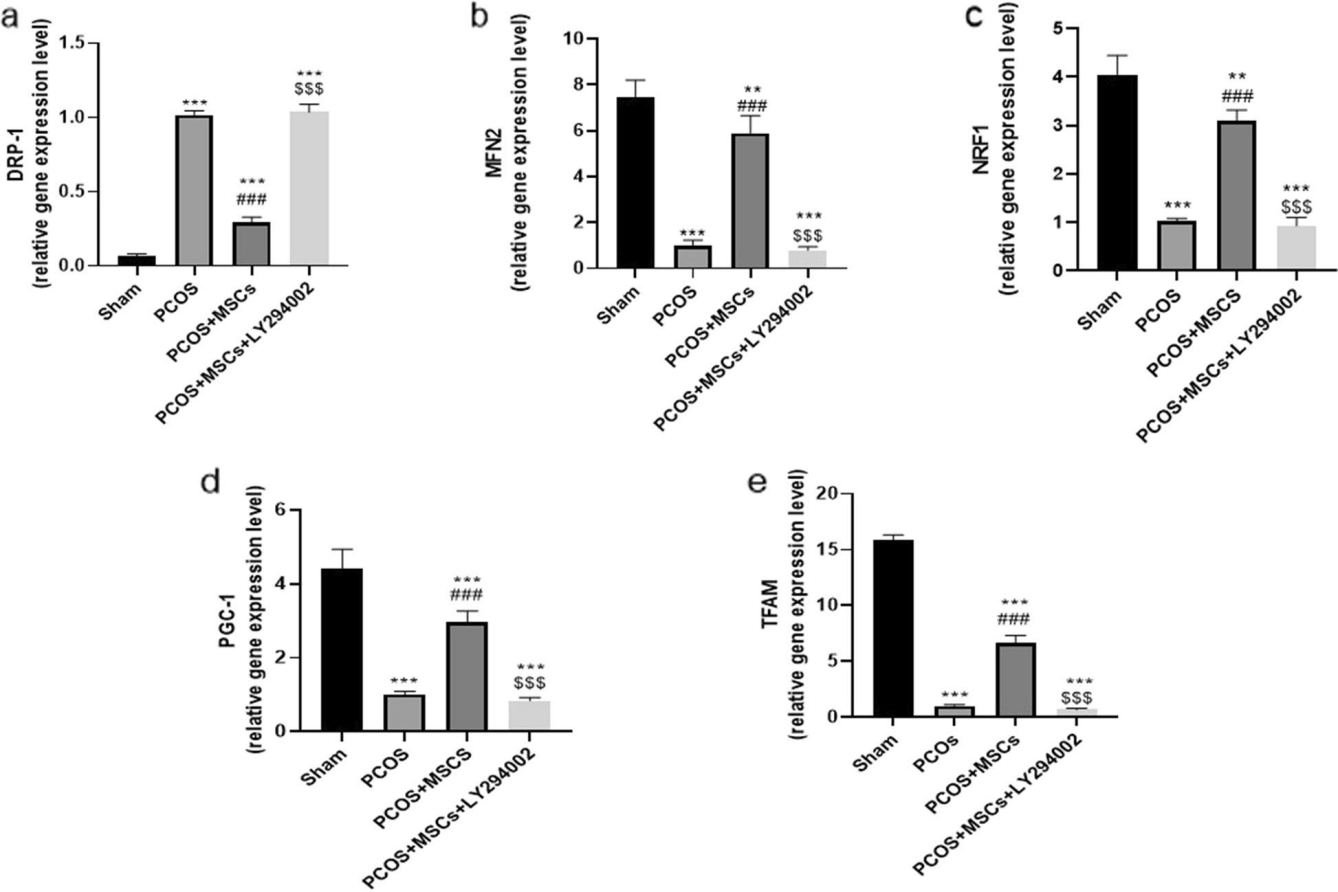
Changes in mitochondrial dynamic markers DRP-1 (**a**), MFN2 (**b**), NRF1 (**c**), PGC1 (**d**) and TFAM (**e**) in different groups. Data are presented as mean ± SEM. ^**^p < . 01 and ^***^p < .001 significant differences versus the Sham group. ^###^*p* < .001 significant differences versus the PCOS group. $$$ *P* < 0.001 significant differences versus the PCOS + MSCs group. (DRP-1: Dynamin-related protein 1, MFN: 2Mitofusin-2, NRF1: Nuclear factor erythroid related factor 1, PGC-1: Peroxisome proliferator-activated receptor-gamma coactivator, TFAM: Transcription Factor A, Mitochondrial)

The PCOS group exhibited marked down regulation in Mfn2, NRF-1, TFAM, PGC1α genes, and a significant up regulation in Drp1 compared to the Sham group **(**Fig. 5, *P* < 0.001). However, the treated rats with MSCs restored the balance in the mitochondrial dynamic genes. LY294002 administration reversed all mentioned parameters in the PCOS + LY294002 groups compared to the PCOS + MSCs group (*P* < 0.001).

**Fig. 6:**
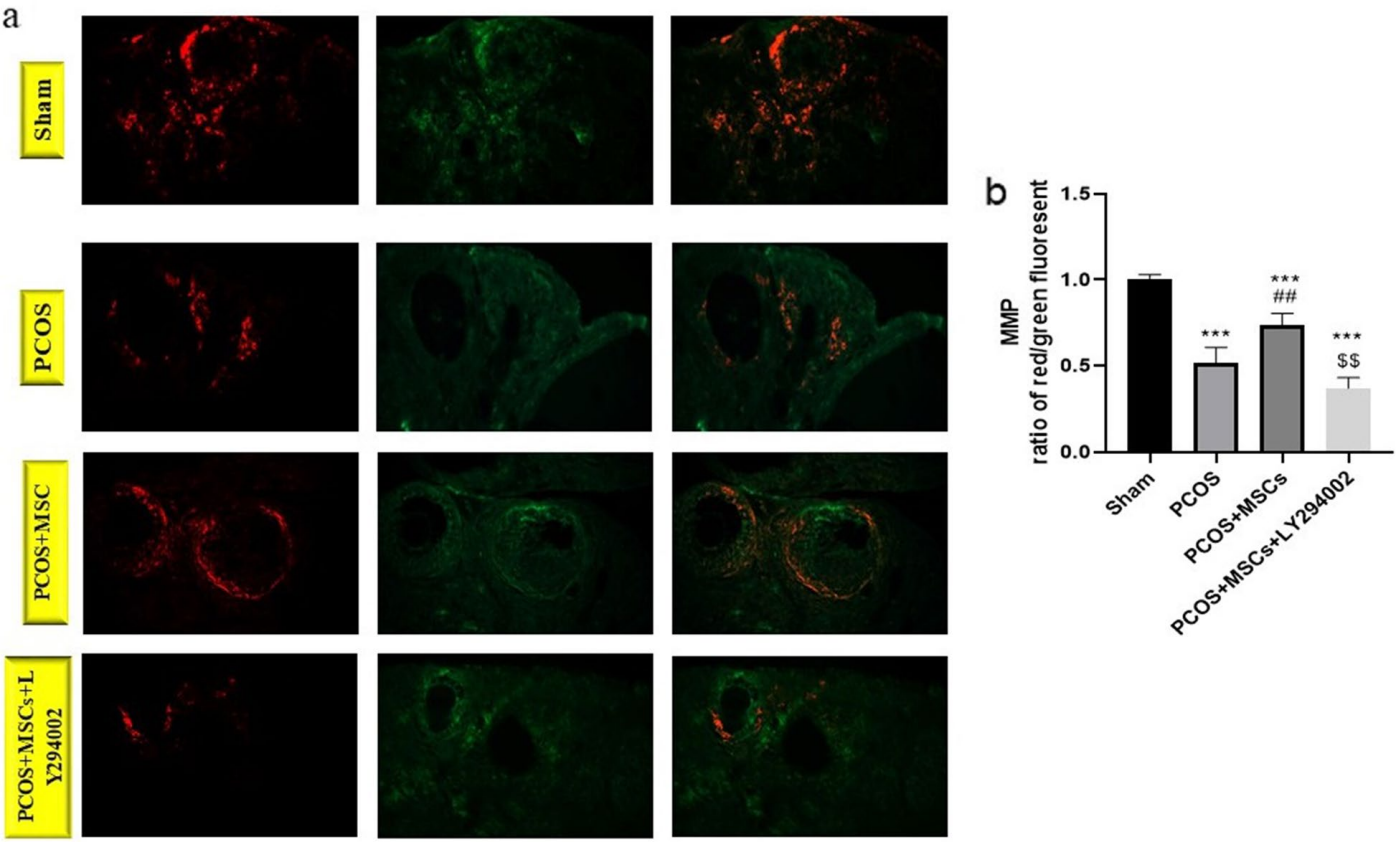
Effect of LY294002 and MSCs on mitochondrial membrane potential. In microscopic fluorescent images, red florescence shows JC-1 aggregating within the mitochondria and in the case of destruction of mitochondria the red color changes into green florescence (**a**), MMP ratio of red/green fluorescent (**b**). Data are presented as mean ± SEM. ^***^p < .001 significant differences versus the Sham group. ^###^*p* < .001 significant differences versus the PCOS group. $$$ *P* < 0. 01, $$, *P* < 0.001 significant differences versus the PCOS + MSCs group

As shown in the Fig. 6, PCOS induction (*P* < 0.001) and LY294002 administration (*P* < 0.05) caused significant decreases in MMP level compared to the Sham group and the PCOS group, respectively. MSC transplantation increased this parameter in comparison with the PCOS group (*P* < 0.001).

**Fig. 7:**
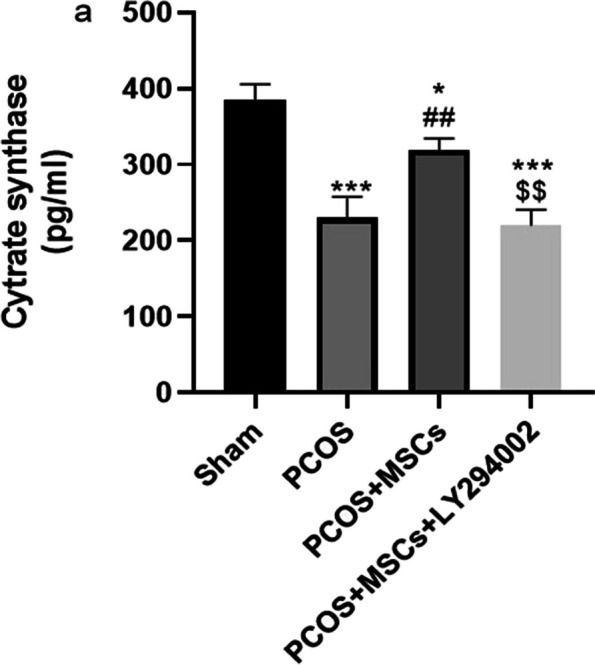
Change in mitochondrial citrate synthase (CS) activity in different groups. Data are presented as mean ± SEM. ^*^p < . 05 and ^***^p < .001 significant differences versus the Sham group. ^##^*p* < .01 significant differences versus the PCOS group. $$ *P* < 0.01 significant differences versus the PCOS + MSCs group

PCOS induction resulted in a notable decrease in ovarian citrate synthase (CS) activity in the PCOS group compared to the Sham group (Fig. 7, *P* < 0.001). The administration of MSCs prevented the reduction of mitochondrial CS activity in the PCOS + MSCs group compared to the PCOS group (*P* < 0.01).

### Effects of adipose-derived MSC and LY294002 administrations on serum hormonal level

**Fig. 8:**
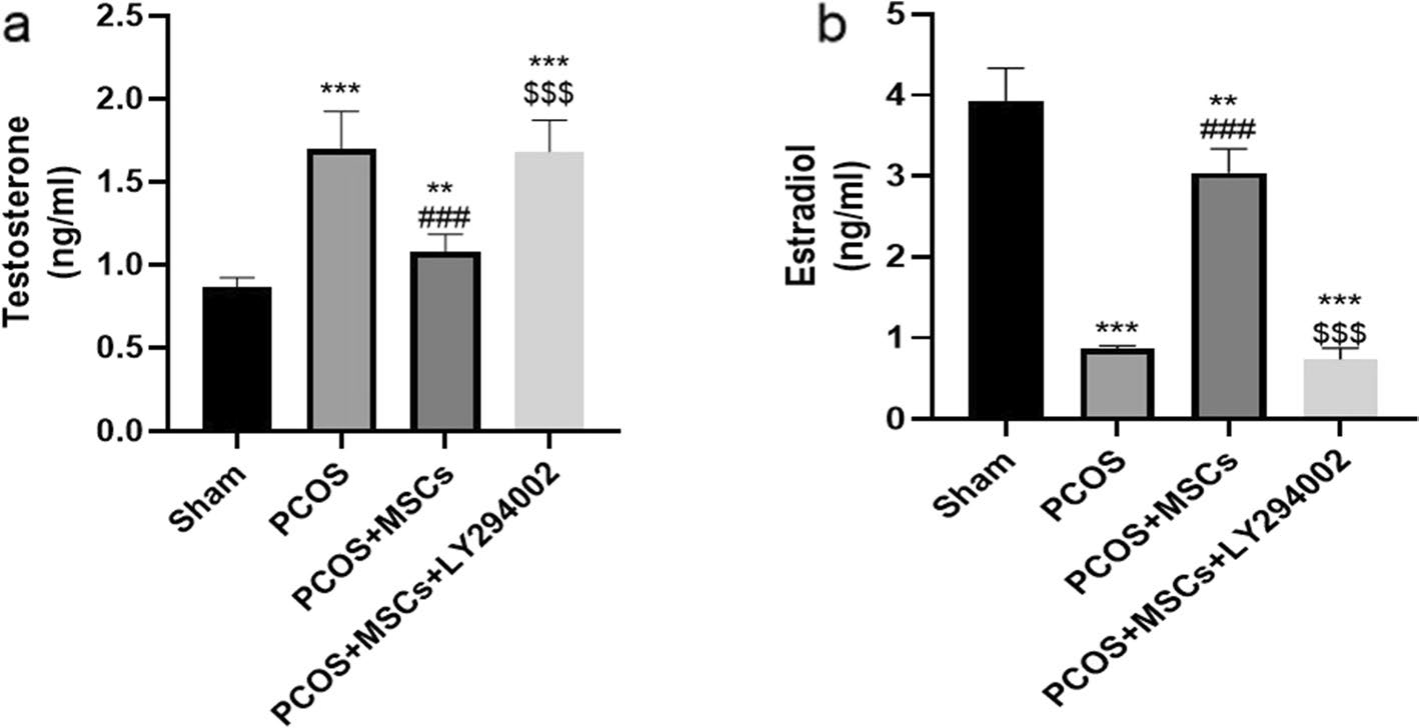
Effects of adipose-derived MSC and LY294002 administrations on serum testosterone and estradiol level. Data are expressed as Mean ± SEM. ^**^p < .01, ^***^p < .001 significant differences versus the Sham group. ^###^*p* < .001 significant differences versus the PCOS group. $$$ *P* < 0.001 significant differences versus the PCOS + MSCs group

As presented in the Fig. 8, the PCOS group showed a notable rise in the serum testosterone level and a remarkable decrease in the serum estradiol level compared to the Sham group (*P* < 0.001). Treatment with MSCs significantly restored these hormonal levels (*P* < 0.001) while LY294002 administration in the PCOS + MSCs + LY294002 group caused a notable increase in the serum testosterone level and a decrease in the serum estradiol level compared to the PCOS + MSCs group (*P* < 0.001).

The results demonstrated that the administration of LY294002 in the Sham + LY294002 and PCOS + LY294002 groups did not lead to any notable alterations in serum testosterone and estradiol levels (Figure S7a and S7b).

### Effects of adipose-derived MSC and LY294002 administrations on serum glucose, insulin level, HOMA-IR and ISI

**Fig. 9:**
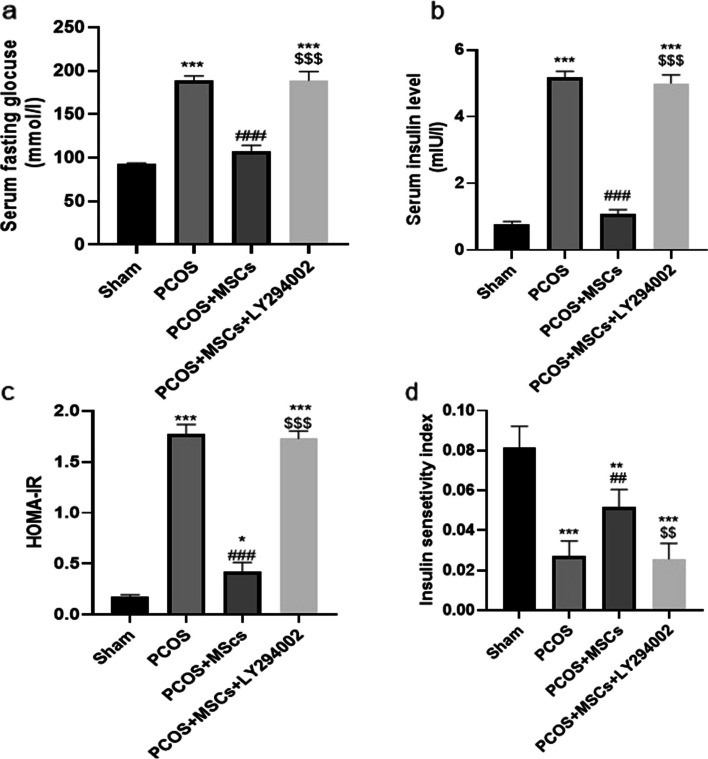
Effects of adipose-derived MSC and LY294002 administrations on serum glucose, insulin level, HOMA-IR and ISI in different groups. Change in serum glucose (**a**), insulin (**b**), HOMA-IR (**c**) and ISI (**d**) in different groups. Data are expressed as Mean ± SEM. ^*^p < .05, ^***^p < .001 significant differences versus the Sham group. ^##^*p* < .01, ^###^*p* < .001 significant differences versus the PCOS group. $$ *P* < 0. 01, $$$ *P* < 0.001 significant differences versus the PCOS + MSCs group

As presented in Fig. 9, the PCOS group manifested a significant increase in the serum fasting glucose, insulin level and calculated HOMA-IR and a decrease in insulin sensitivity index (ISI) as compared to the Sham group (*P* < 0.001). After administration of MSCs, the PCOS + MSCs group showed significant decreases in the serum fasting glucose, insulin level and calculated HOMA-IR (*P* < 0.001) but a considerable increase in ISI (*P* < 0.01). LY294002 administration caused significant increases in the serum fasting glucose concentration, insulin level and HOMA-IR (*P* < 0.001) and a decrease in ISI (*P* < 0.01) in the PCOS + MSCs + LY294002 group compared to the PCOS + MSCs group.

### Effects of adipose-derived MSC and LY294002 administrations on PI3K/AKT pathway activation

**Fig. 10:**
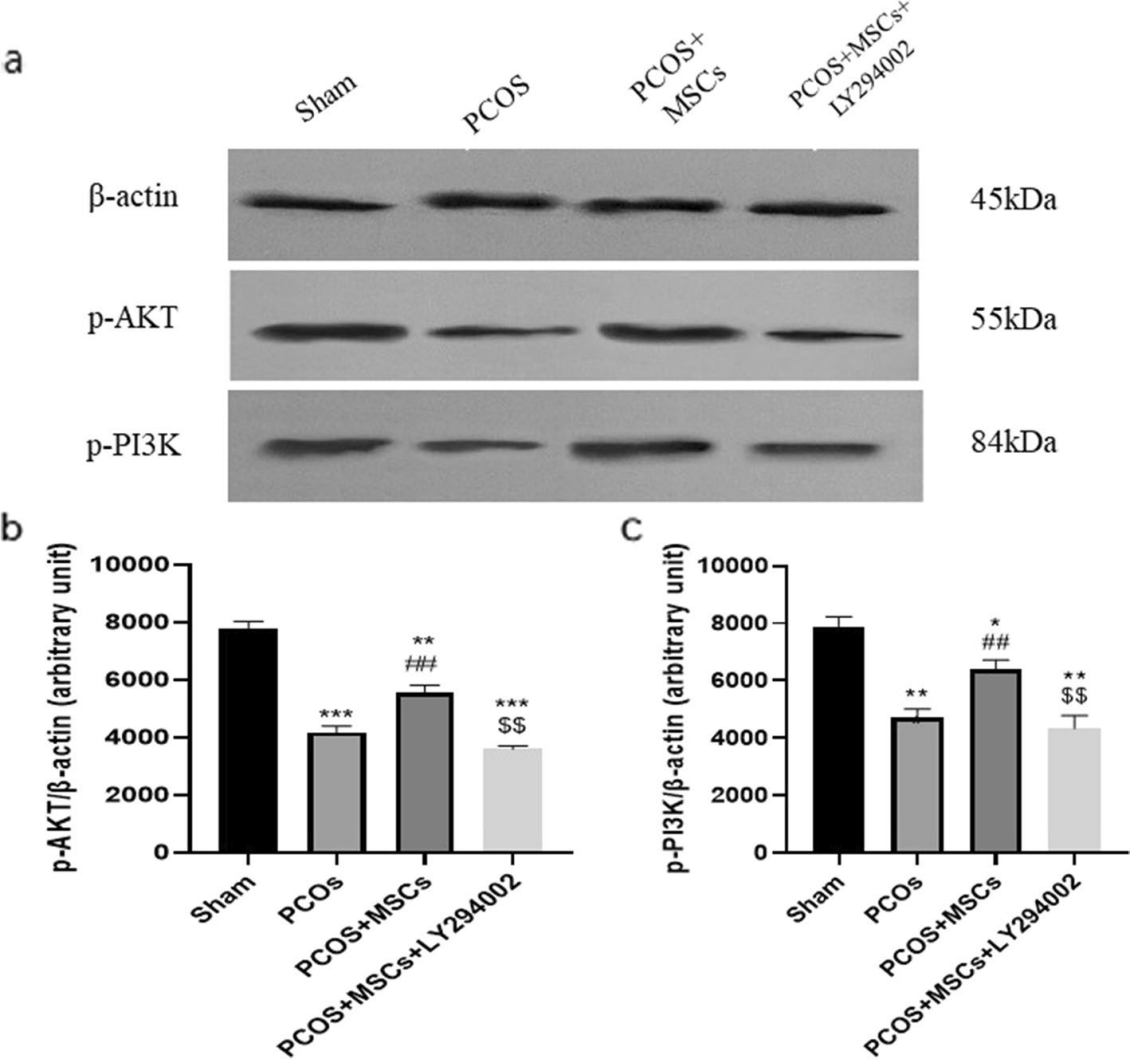
pAKT, pPI3K and β-actin changes in ovarian tissue in different groups. Data are presented as means ± SEM. * *P* < 0.05, ** *P* < 0.01. *** *P* < 0.001 significant differences versus the Sham group, ## *P* < 0.01 significant differences versus the PCOS group. $$ *P* < 0.01 significant differences versus the PCOS + MSCs group. $$ *P* < 0. 01, $$$ *P* < 0.001 significant differences versus the PCOS + MSCs group

To assess the mechanisms through which MSCs improved PCOS changes in more detail, we next measured P-PI3K and P-AKT protein levels via the western blotting. Our results revealed that the animal model of PCOS exhibited significant decreases in phosphorylated form of these proteins compared to the Sham group (Fig. 10, *P* < 0.001). MSCs administration caused significant increases in P-AKT and P-PI3K protein levels compared to the PCOS group (*P* < 0.01). However, animals which received inhibitor of P-PI3K showed downregulation of P-AKT and P-PI3K protein levels compared to the PCOS + MSCs group (*P* < 0.01).

## Discussion

Based on recent studies, a defect in mitochondrial dynamic (by fission and fusion) and biogenesis could lead to consequences such as insulin resistance, hyperandrogenism, oxidative stress and glucose intolerance which are considered key features in PCOS [29] as well as female infertility [45]. Restoring mitochondrial dynamics and promoting mitochondrial biogenesis by mitigating oxidative stress could lead to ameliorating polycystic ovary syndrome [46] and are crucial for maintaining healthy follicular development [47]. Mitochondrial fission is regulated by expression levels of Drp1 gene, while mitochondrial fusion is mediated by expression levels of MFN2 genes. Mitochondrial biogenesis is modulated by the expression levels of genes such as PGC-1α, NRF1 and TFAM [48]. The study conducted by XIE et al., demonstrated that expression level of mentioned genes altered within the ovaries of patients with PCOS compared to the control group. Furthermore, this research has suggested that ameliorating ovarian mitochondrial function (including dynamic and biogenesis) could improve the quality of oocyte and follicular development in patients with PCOS. Therefore, mitochondrial fission, fusion and biogenesis genes could be key players in clinical outcome of PCOS patients [49]. Our findings, as demonstrated by JC-1 staining, revealed a reduction in mitochondrial membrane potential (MMP) in PCOS rats. This reduction in MMP can lead to substantially decreased efficiency of oxidative phosphorylation and respiratory chain-mediated proton pumping from the matrix, which can account for reactive oxygen species (ROS) production and various mutants in mitochondrial genes and malfunction, as well as damage to oocyte development potential [50]. MSCs administration restored mitochondrial dynamic balance, increased CS activity and MMP. MSCs, with their specific features, could rescue damaged ovarian tissue and cells, possibly by transferring their own mitochondria through membrane thin channels [51]. Mitochondrial transfer restores oxidative phosphorylation, dynamic balance, MMP and CS activity. One study demonstrated that a calcium-binding mitochondria Rho GTPase is a key mediator of MSC-derived organelle that enables the movement of mitochondria along microtubules [52]. Furthermore, the transplantation of MSCs has the potential to regulate the balance of mitochondrial dynamics through posttranslational alterations to Drp1 and enhance mitochondrial fission.

PCOS often appears with insulin resistance (IR) and hyperandrogenism [53]. IR can cause overproduction of androgen levels, which increases the risk of cardiovascular diseases, tumors and metabolic disorders [54]. In our study the concentration of testosterone, insulin levels and serum glucose levels were elevated in PCOS rats. PCOS induction led to ovarian hyperandrogenism and significantly increased basal insulin secretion. The occurrence of IR and hyperandrogenism is crucial for mitochondrial malfunctions to impair follicular development and progression of PCOS [55]. Mitochondria plays a key role in normal insulin function, and conversely, insulin secretion is an important mediator of oxidative phosphorylation and mitochondrial function [56]. In addition to the higher serum glucose, elevated HOMA-IR and lower ISI in our results, mitochondrial dysfunction led to IR, excessive androgens and oxidative stress. The decrease in HOMA-IR, insulin level and glucose concentration, as well as an increase in ISI, were observed with the MSCs transplantation via their antioxidant effects. This positive effect may rescue the function of mitochondria, leading to improvements in these parameters. In the present study, the induction of PCOS and inhibition of the PI3K-AKT pathway caused a reduction of estradiol and an enhancement of testosterone concentration. MSCs treatment significantly increased estradiol and decreased testosterone concentration in the PCOS + MSCs group. Several studies, including Park et al., have demonstrated that MSCs transplantation could decrease androgen synthesis pathways through paracrine secretion via exosomes [57].

In our study, MSCs transplantation restore the oxidant anti-oxidant balance. The induction of PCOS caused significant elevation in MDA levels, which can affect oocyte differentiation. Elevated MDA levels may cause a decrease in ISI and an increase in HOMA-IR, as well as decreased glucose uptake in insulin-sensitive cells and increased blood glucose concentration [56]. One study reported that MSCs, with their secretion of HSPs and regulation of free calcium and ATP in mitochondria, can rescue oxidative stress conditions. Therefore, HSPs secreted from MSCs could lead to phosphorylation of intracellular tyrosine kinase and activation of PI3K-AKT pathway to restore oxidative balance [58]. The pathogenesis of PCOS is significantly influenced by the inflammatory response, which can cause metabolic irregularities, deteriorate oxidative stress status and ovarian dysfunction in PCOS patients [59]. It has been demonstrated in recent studies that PCOS patients have elevated levels of inflammatory markers, such as TNF-α and IL-6 [31, 60]. Consistent with previous studies on this case, we demonstrated that induction of PCOS could increase inflammatory markers such as TNF-α and IL-6 in ovarian tissue. In this study, we demonstrated that administration of MSCs could regulate inflammation by decreasing the levels of two pro-inflammatory factors, including TNF-α and IL-6. The current findings suggested that MSCs have anti-inflammatory effect, capable of converting a pro-inflammatory to an anti-inflammatory state and reduced the pathological changes in local ovarian tissue of PCOS rats.

Letrozole is a non-steroidal aromatase inhibitor that blocks the conversion of testosterone to estradiol, leading to increased levels of androgens in the blood and ovaries [61]. The PCOS model induced by letrozole exhibited various facets of PCOS, including reproductive and metabolic features, making it a valuable model for PCOS studies [62]. The Rotterdam Criteria define PCOS by the presence of at least two of these three conditions: 1- elevated androgen levels, either clinically or biochemically, 2- oligo- or amenorrhea, and 3- polycystic ovaries [63]. Considering the fact that we observed a significant increase in serum testosterone levels and an increase in the number of cystic follicles in the PCOS group compared to the sham rats, PCOS model was certainly established. Zhang et al., have shown that overproduction of ROS (reactive oxygen species) in mitochondria can lead to an increase in atresia of antral follicles, corpus luteum, preovulatory and graafian follicles. Additionally, increased levels of ROS have been shown to cause ovarian cell mitophagy and destruction [56]. In present study, our results showed an elevation in pre-antral and cystic follicle, while there was a reduction in antral, preovulatory and graafian follicles, which may be related to mitochondrial dysfunction. It seems that MSCs restore follicular development by inhibiting oxidative stress and improving mitochondrial function. There is little controversy about the differentiation of mesenchymal stem cells, and the mentioned feature in the introduction is one of the general effects of mesenchymal stem cells [64]. Recent research indicated that the positive effects on tissue repair following mesenchymal stem cell transplantation resulted from releasing soluble factors through the paracrine mechanism [65]. In our recent studies, MSCs transplantation via intraperitoneal injection have shown that MSCs may have beneficial therapeutic effects in septic shock and cerebral ischemia reperfusion, primarily through paracrine pathways [20, 66, 67]. Several studies, using animal model and transitional research, have demonstrated that MSCs could home to sites of injury, such as the ovary in PCOS. Furthermore, studies have shown that the positive effects of MSCs on ovarian function are mainly exerted via the paracrine pathway [68, 69].

Recent studies have widely reported that the PI3K/AKT signaling pathway is dysregulated in both patients with PCOS and animal models of PCOS, additionally the critical role of the PI3K-AKT signaling pathway in regulating of the growth, development, differentiation, and survival of ovarian follicles were documented [12, 70]. Therefore, maintaining optimal PI3K-AKT signaling pathway activity appears to be essential for the normal development and physiological functions of the ovary, as a result, disruption of this pathway plays an important role in the pathogenesis of PCOS [70]. Dysfunctional PI3K-AKT pathway has been associated with insulin resistance, anovulation, decreased granulosa cell proliferation, reduced number of mature follicles, increased atresia follicles, and ultimately results in decreased fertility [71]. Previous studies have recommended further research on the PI3K-AKT pathway and its inhibitor (LY294002), that not only enhance understanding of the pathogenesis of PCOS but also offer a novel approach so as to treat PCOS [72]. In the present manuscript, the administration of LY294002 as a PI3K-AKT pathway inhibitor could exacerbate the pathogenesis of PCOS by diminishing the survival and differentiation of ovarian tissue. Our study exhibited that activation of the PI3K-AKT signaling pathway is primary pathway for preserving follicles survival and determining female reproductive life span. Our results revealed the protein expression of pAKt and pPI3K was decreased in PCOS rats. Several studies have confirmed PI3K-AKT signaling pathway key role in insulin resistance and have shown MSCs are involved in the activation of this intracellular signaling pathway [6, 27]. It was also reported that ovarian cells proliferation and apoptosis are regulated by PI3K-AKT pathway [27]. In our study, MSCs transplantation led to an increase in protein expression of pAKT and pPI3K. The ability of MSCs transplantation to enhance the activation of the PI3K-AKT signaling pathway could improve the pathogenesis of PCOS and insulin resistance. Additionally, to support the role of PI3K-AKT pathway in MSCs function, a PI3K-AKT inhibitor (LY294002) was applied. Interestingly, in the PCOS + MSCs + LY294002 group, all parameters deteriorated compared to the PCOS + MSCs rats, indicating that inhibition of the PI3K-AKT signaling pathway completely blocked the beneficial effects of MSCs on PCOS ovaries. These findings demonstrate the effectiveness of MSCs in ameliorating PCOS ovaries is mainly attributed to the PI3K-AKT pathway and paracrine mechanism. Notably, the PI3K/AKT signaling pathways are recognized for their pivotal role in regulating multiple cellular behavior of MSCs, such as survival, proliferation, growth and mobilization [73, 74]. Huang et al., demonstrated that inhibition of PI3K-AKT pathway with LY294002 could disrupt intracellular homeostasis by inducing mitochondrial dysfunction [73]. Recent studies indicate that disruption of the PI3K-AKT pathway can lead to hypoxia and apoptosis in MSCs [75]. Inhibiting this pathway with LY294002 could potentially reverse the protective effects of MSCs and affect the transfer and restoration of mitochondria [76]. Therefore, optimal activity of the PI3K-AKT signaling pathway is essential for better outcomes in stem cell therapy [77]. By regulating the status signaling using LY294002, we exhibited that inhibiting the PI3K-Akt signaling pathway reduced the expression of mitochondrial dynamic genes, MMP and CS activity. Thus, we suggest this pathway may be related to mitochondrial dysfunction and the pathogenesis of PCOS. In accordance with previous studies, in our study there was no significant difference in all parameters between the Sham and Sham + LY294002 groups [78]. In this study, no notable difference was observed between the PCOS + MSCs + LY294002 and PCOS + LY294002 groups. Recent studies have shown that the LY294002 could affect the activity and survival of MSCs. It effectively prevented the impact of MSCs on ovaries [79]. The administration of LY294002 leads to increased ROS production [80], raised apoptosis [81, 82], reduced proliferation [83], decreased the gene expression of cyclin D1 and E1 and finally diminished the activity of MSCs [84]. One of the limitations of the present study was the lack of localization of MSCs in ovarian tissue. It is also recommended to investigate the effect of MSCs on apoptosis and autophagy from the PI3K-AKT pathway in future studies.

## Conclusion

**Fig. 11:**
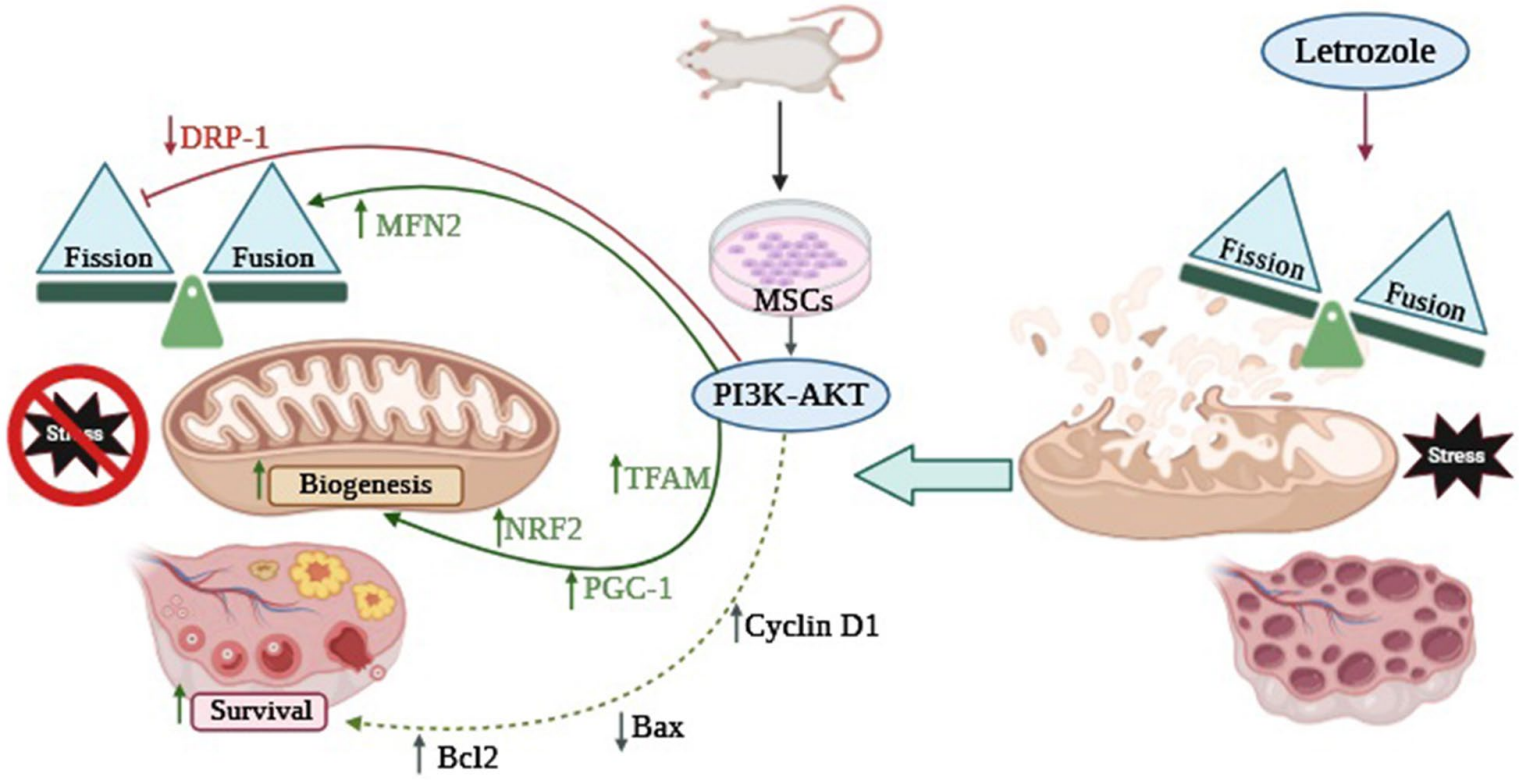
Hypothetical schematic model showing letrozole administration on mitochondrial dynamics, oxidative stress and ovarian morphology and the effects of MSC transplantation through the PI3K-AKT pathway in PCOS

Our study showed that the protective effects of MSC transplantation in regulating mitochondrial dynamics, promoting mitochondrial biogenesis, improving ovarian histological changes, and competing with redox status were mainly mediated through the PI3K-AKT pathway in the PCOS model (Fig. 11).

### Supplementary Information

**Supplementary Material 1. **

## Data Availability

The datasets/information used for this study is available on reasonable request.

## Abbreviations

MSCs: Mesenchymal stem cells
PCOS: Polycystic Ovary Syndrome
MMP: Mitochondrial membrane potential
CS: Citrate synthase
HOMA-IR: Homeostatic model assessment for insulin resistance
SOD: Ovary superoxide dismutase
MDA: Malondialdehyde
PI3K-AKT: Phosphoinositide-3-kinase-protein kinase B/AKT
WHO: World Health Organization
TBA: Thiobarbituric acid
PBS: Phosphate-buffered saline
FBS: Fetal bovine serum
NRF1: Nuclear factor erythroid 2–related factor 1
MFN2: Mitofusin 2
DRP-1: Dynamin-related protein 1
PGC-1: Peroxisome proliferator-activated receptor-γ coactivator
TFAM: Mitochondrial transcription factor A
