# Supplementary material for: The effect of adipose-derived mesenchymal stem cell transplantation on ovarian mitochondrial dysfunction in letrozole-induced polycystic ovary syndrome in rats: the role of PI3K-AKT signaling pathway
Source: J Ovarian Res. 2024 Apr 27;17:91. doi: 10.1186/s13048-024-01422-3 (PMC11056058; doi:10.1186/s13048-024-01422-3)
Supplement: Supplementary file 1 — Supplementary Material 1. [file 13048_2024_1422_MOESM1_ESM.docx]

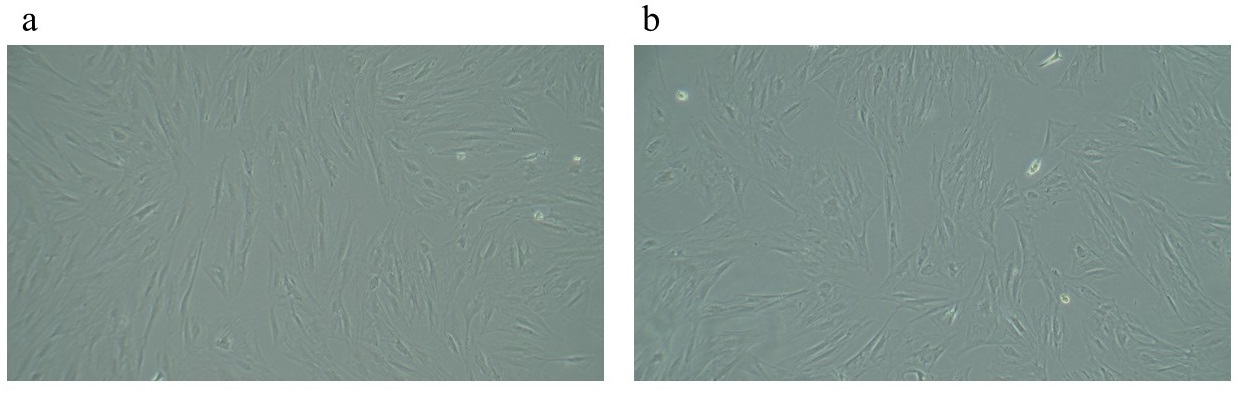


**Figure S1**. The attachment of mesenchymal stem cells derived from adipose tissue of Wistar rats were mostly spindled (fibroblastic morphology) in the first (a) and second passages (b).


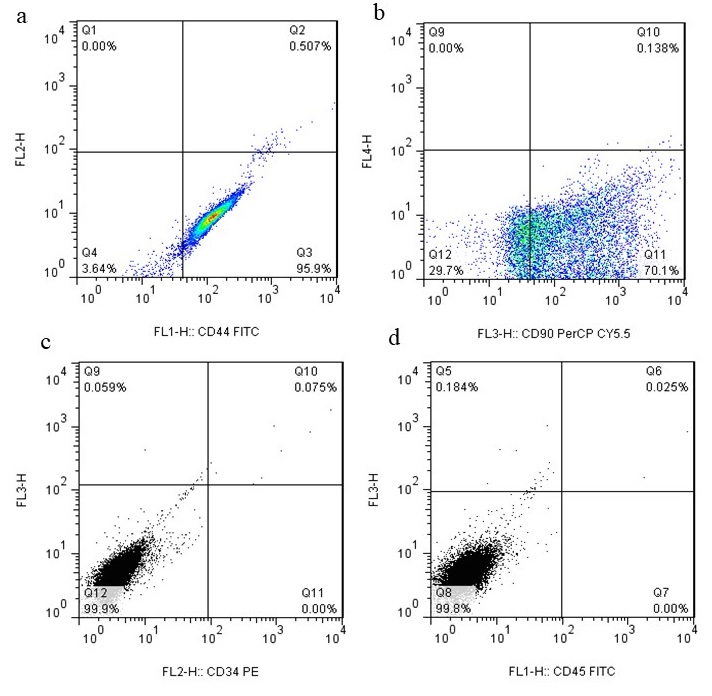


**Figure S2.** Characterization of rat adipose tissue-derived MSCs by flow cytometry analysis. Figure (a) and (b) show MSCs with high expression of mesenchymal markers [CD44 and CD90]. Figure (c) and (d) demonstrate low hematopoietic markers [CD34 and CD45).


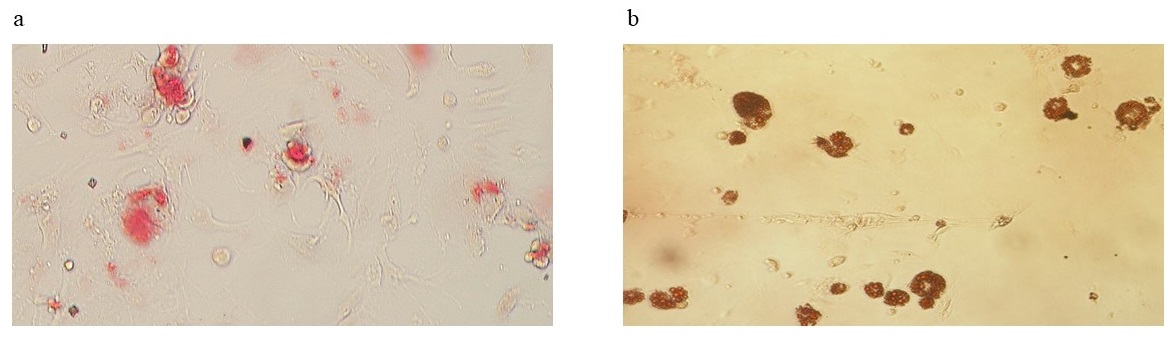


**Figure S3.** Areas exhibiting osteogenic activity were visualized as red regions using the Alizarin Red staining method (a). Lipid droplets that formed as a result of adipogenic differentiation in the cells were stained red using the Oil Red staining method (b).


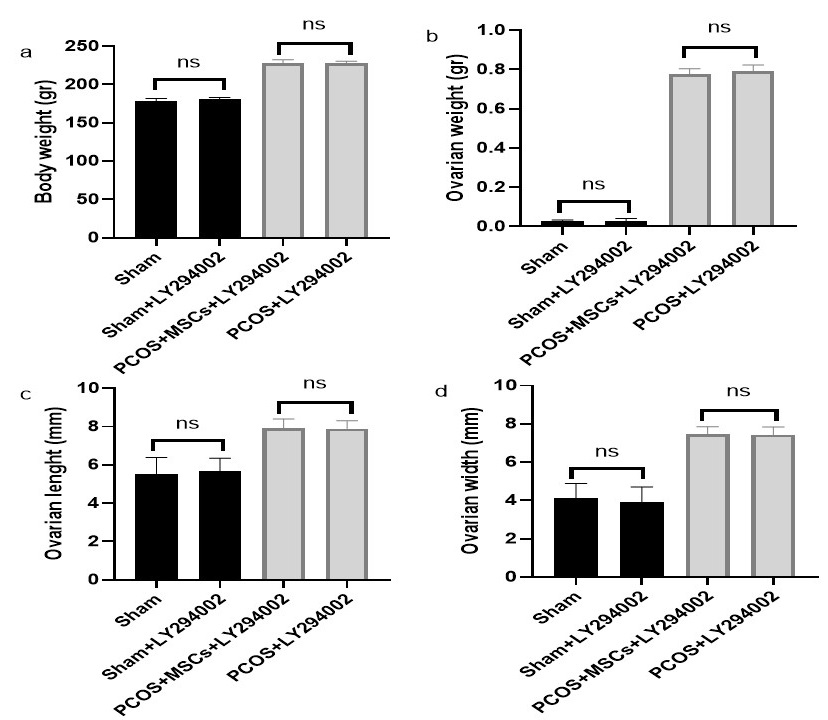


**Figure S4.** Changes in body weight (a), ovarian weight (b), ovarian length (c) and ovarian width (d) among the different groups**.** There was no significant difference in the mentioned parameters between the sham versus Sham+LY294002 and PCOS+MSCs+LY294002 versus PCOS+LY294002 groups.


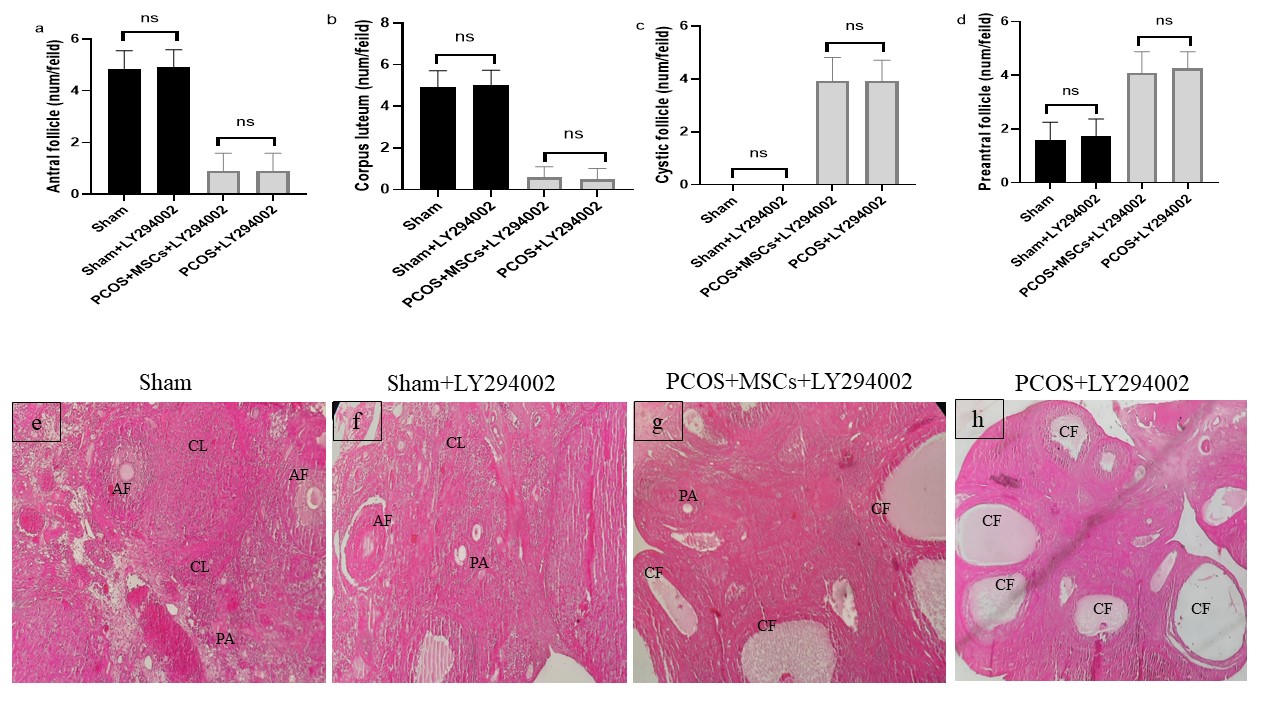


**Figure S5.** Histopathological examination of ovarian tissue. antral follicle (a), corpus loteum (b), cystic follicle (c), preantral follicle (d), Sham group (e), Sham+LY294002 (f), PCOS+MSCs+LY294002 (g) and PCOS+LY294002 (h). The mentioned follicular parameters did not show significant differences between the sham and Sham+LY294002 groups, as well as between the PCOS+MSCs+LY294002 and PCOS+LY294002 groups.


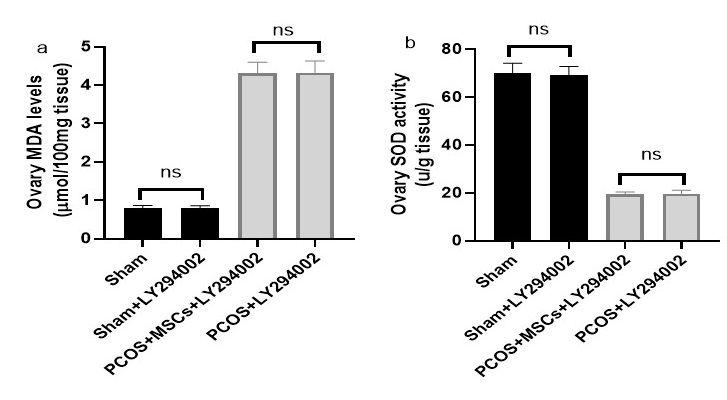


**Figure S6.** Changes in ovarian MDA level (a) and SOD activity (b) among the different groups. There was no significant difference in the MDA level and SOD activity between the Sham versus Sham+LY294002 and PCOS+MSCs+LY294002 versus PCOS+LY294002 groups.


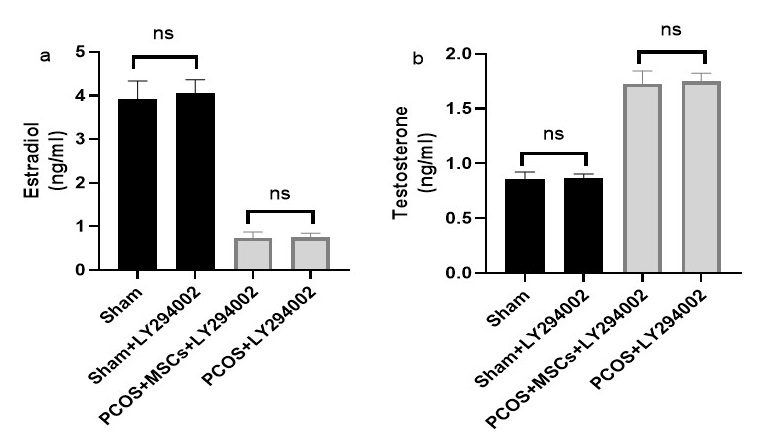


**Figure S7.** The study analyzed changes in ovarian estradiol (a) and testosterone levels (b) across the various groups, and discovered that there was no significant difference in the estradiol and testosterone levels between the Sham and Sham+LY294002 groups, as well as between the PCOS+MSCs+LY294002 and PCOS+LY294002 groups.
